# Associations of multicultural status with depressive mood and suicidality among Korean adolescents: the roles of parental country of birth and socioeconomic position

**DOI:** 10.1186/s12889-017-4044-y

**Published:** 2017-01-25

**Authors:** Jinwook Bahk, Agnus M. Kim, Young-Ho Khang

**Affiliations:** 1 Institute of Health Policy and Management, Seoul National University Medical Research Center, 103 Daehak-ro, Jongno-gu, Seoul 03080 South Korea; 2 Department of Health Policy and Management, Seoul National University College of Medicine, 103 Daehak-ro, Jongno-gu, Seoul 03080 South Korea

**Keywords:** South Korea, Immigrant, Adolescent, Mental health, Depressive mood, Suicidality, Socioeconomic position

## Abstract

**Background:**

The mental health of the offspring of immigrants is a major public health concern. In this study, we examined associations of multicultural status and parental country of birth with adolescent mental health in South Korea, and assessed the effect of socioeconomic position (SEP) on these associations.

**Methods:**

We used four waves of the Korea Youth Risk Behavior Web-based Survey (KYRBS) between 2011 and 2014, including 294,324 participants (149,219 boys and 145,105 girls aged 13–18 years) as study subjects. KYRBS is a cross-sectional survey conducted annually by the Korea Centers for Disease Control and Prevention. The participants in the KYRBS were drawn as stratified multistage clustered samples from Korean middle schools and high schools. We calculated the age-adjusted 12-month prevalence of depressive mood and suicidal behaviors by parental country of birth, and estimated the effects of SEP indicators on the relationship.

**Results:**

The age-standardized prevalence of suicidality (suicide ideation, plans, and attempts) was significantly different between multicultural and non-multicultural boys. The impact of multicultural status on mental health varied with parental foreign-born status and maternal country of birth. Compared with non-multicultural counterparts, boys with Japan-born mothers showed lower prevalence ratios (PRs) of suicidal plans (*PR* = 0.34, 95% CI 0.16–0.70). Girls with Japan-born mothers also showed lower PRs of depressive mood (*PR* = 0.77, 95% CI 0.63–0.95) and suicidal ideation (*PR* = 0.59, 95% CI 0.41–0.83), while adolescents with Korean-Chinese mothers showed similar PRs. Boys with foreign-born fathers as well as boys with two foreign-born parents were at a greater risk of suicidality than non-multicultural boys. The magnitude of the relationship between multicultural status and mental health outcomes was generally attenuated after adjusting for SEP indicators.

**Conclusions:**

In general, adolescents with Japan-born mothers showed lower PRs of depressive mood and suicidality than non-multicultural adolescents, while those with Korean-Chinese mothers showed similar PRs. Boys who had foreign-born fathers generally showed greater PRs of depressive mood and suicidality than non-multicultural boys. To ensure the effective implementation of policies to reduce mental health problems among multicultural adolescents in South Korea, detailed information should be considered regarding the cultural and socioeconomic backgrounds of families, such as parental country of birth and SEP.

**Electronic supplementary material:**

The online version of this article (doi:10.1186/s12889-017-4044-y) contains supplementary material, which is available to authorized users.

## Background

The mental health of the offspring of immigrants has been recognized as a major public health concern in many countries [1–6]. Studies have reported increased risks of mental health problems such as depression, psychiatric disorders, and suicidal behavior among the offspring of immigrants [2, 3, 7–9]. However, most studies on the issue have been performed in Western countries. Research on the mental health of multi-ethnic or multi-national adolescents in Korea has only been initiated recently [10–14].

While international migration has become a global phenomenon over recent decades, South Korea (hereafter ‘Korea’) has experienced this phenomenon relatively recently. The registered foreign population in 1993 was only 76,374, comprising 0.17% of the total population of Korea, but increased to nearly one million in 2013, accounting for 1.9% of the total population [15] (see Additional file 1: Figure S1). This change is partially due to an increase in the number of international marriages. Among the foreign population in 2013, 16% were marriage migrants, which were the second largest group, following migrant workers [16]. The Korean government started to use the term, ‘multicultural family’, as the umbrella term for migrants (including marriage migrants, migrant workers, North Korean defectors, and Korean-Chinese) and their families in Korea. Based on the information provided by Statistics Korea about marriage registration, approximately one in ten marriages in 2010 (10.8%) involved international couples and 74.6% of the international marriages were between a Korean groom and foreign bride. Chinese were the majority of marriage partners (40.8%) of all female spouses; Vietnamese women were second (34.0%), followed by Filipino women (6.9%) [17, 18]. With the increase in international marriage, children with multicultural backgrounds or multi-ethnicity (hereafter referred to as ‘multicultural adolescents’) have recently become a meaningful part of the population of young people in Korea, which account for 2% of the total Korean population under 18 years of age in 2014 [16]. At the same time, the health and well-being of multicultural adolescents became an important research question.

Factors hypothesized to increase the risk of mental health problems in this population include low language competence, differences in external appearance from the majority population, acculturative stress, and discrimination associated with the above factors, as well as the social challenges that are frequently faced by the offspring of immigrants, such as poor family functioning and low socioeconomic position (SEP) [2, 6, 19]. In addition, mental health problems among multicultural adolescents may be heterogeneous across ethnic subgroups, meaning that a simple comparison between multicultural and non-multicultural adolescents may be insufficient. For example, among Asian-American adolescents in the USA, the levels of psychological symptoms and suicidal behaviors have been found to differ among ethnic subgroups such as Koreans, Japanese, Chinese, and Southeast Asians [20, 21]. Some of the observed differences in health metrics among various ethnic groups might be partly due to the effect of SEP [22–24]. Among adolescents from four main cultural subgroups in the USA (African-American, Native American, Asian-American, and Latino), each subgroup showed intragroup differences in risk for suicidal behaviors according to SEP and ethnic background [23]. Moreover, the level of perceived discrimination may vary across ethic groups and country of birth [19, 25–27]. For instance, a UK study showed that the prevalence of perceived discrimination experiences differed across the ethnic groups: the Black Caribbean was more likely to be exposed to the unfair treatment, while Indian and Bangladeshi reported higher levels of insults than UK whites [26].

In this study, we explored associations between mental health (measured with questions on depressive mood, suicidal ideation, suicidal plans, and suicide attempts) and multicultural status (defined by parental country of birth), and examined the effect of SEP on these associations. Exploring adolescent mental health patterned by parental country of birth and the role of SEP in the association of multicultural status with mental health is likely to lead to a better explanation of the nature of discrimination, which is a serious problem faced by the multicultural families of Korea [28, 29]. We used nationally representative data of Korean middle-school and high-school adolescents collected between 2011 and 2014. Our hypotheses were as follows: First, multicultural adolescents were hypothesized to be at a greater risk for mental health problems than non-multicultural adolescents; Second, the impact of multicultural status on mental health was hypothesized to vary with parental foreign-born status and parental country of birth; Third, the relationship between mental health and multicultural status was hypothesized to be partly explained by SEP; in particular, the magnitude of the relationship between multicultural status and poor mental health was hypothesized to be attenuated by adjustment of SEP.

## Methods

### Data

Data were obtained from four waves of the Korea Youth Risk Behavior Web-based Survey (KYRBS), conducted from 2011 to 2014 [30]. KYRBS is to assess health-risk behaviors, including tobacco and alcohol use, obesity and weight control effort, physical activity, dietary behaviors, injury, sexual behaviors, and mental health, among middle- and high-school students in Korea. Questions about multicultural status were first included in 2011, and have subsequently been included in each wave of KYRBS. KYRBS is a repeated cross-sectional online survey administered to a nationally representative sample of Korean middle-school and high-school students aged 13–18 years, and is annually conducted by the Korea Centers for Disease Control and Prevention with administrative cooperation of the Korean Ministry of Education. The participants in KYRBS were drawn as stratified multistage clustered samples from Korean middle schools and high schools. A reliability test of the KYRBS questionnaire was conducted in 2008 for 2,298 middle and high school students with the two surveys being about 2 weeks apart. For suicide ideation and suicide attempt, the Kappas were 58% and 70%, respectively, and the percent agreements were 87.3% and 88.1%, respectively [31]. The number of study subjects who completed the questionnaire was 75,643 (from 800 schools) in 2011; 74,186 (from 797 schools) in 2012; 72,435 (from 799 schools) in 2013; and 72,060 (from 799 schools) in 2014. A total of 294,324 participants (149,219 boys and 145,105 girls) were analyzed. The participant response rate for KYRBS was very high, ranging from 95.5% in 2011 to 97.2% in 2014. The school response rates for KYRBS ranged from 100% in 2011 to 99.9% in 2014. Additional details about KYRBS are available elsewhere [30].

### Outcomes

In KYRBS, five mental health indicators were included. One question on the seriousness of suicide attempt (medical treatment after a suicide attempt) was not used in this study, due to small cell counts. We included depressive mood and three suicide-related metrics (suicidal ideation, suicidal plans, and suicide attempts) as mental health indicators. Depressive mood was assessed using the following question: “During the last 12 months, did you have feelings of sadness or despair that have interrupted your daily life for at least 2 weeks?” Suicidal ideation was measured with the following question: “During the last 12 months, have you ever seriously contemplated suicide?” Suicidal plans were assessed based on the following question “During the last 12 months, have you ever made specific plans for suicide?” Suicidal attempts were assessed by the following question: “During the last 12 months, have you ever attempted suicide?” The response categories for these four questions were yes or no.

### Multicultural status

We used parental country of birth as an indicator for multicultural status of adolescents, since the offspring of migrants might be more likely raised in multicultural environments. Multicultural status was dichotomized as non-multicultural (both parents born in Korea) or multicultural (at least one parent born in a foreign country). North Korean defectors and Korean-Chinese (ethnic Koreans born in China) were also considered as foreign-born parents. In order to identify more detailed sub-group differences, multicultural status of adolescents was further subdivided into five categories: adolescents with Japan-born mothers and Korean-born fathers; with Korean-Chinese mothers and Korean-born fathers; with other foreign-born mothers and Korean-born fathers; with foreign-born fathers and Korean-born mothers; or with two foreign-born parents. The offspring of Japan-born mothers and Korean-Chinese mothers were the two major sub-groups; therefore, these were the only two groups that we were able to create as a separate multicultural group. While the other groups included parents born in a range of countries (see Additional file 1: Table S1). Information on each adolescent’s country of birth and length of stay in Korea was not available in KYRBS.

### Socioeconomic position indicators

We used six SEP indicators; urbanity (metropolitan cities, small and medium cities, and rural areas), maternal and paternal education level (middle school or lower, high school, college or higher, do not know, and no parent), perceived household economic status (high, mid-high, middle, mid-low, and low), self-reported educational performance (high, mid-high, middle, mid-low, and low), and cohabitation with parents (living with both father and mother, living with only father, living with only mother, and living with neither father nor mother) [32]. Perceived household economic status was measured by asking the participant, “What is your household economic status?” Self-reported educational performance was based on the question, “During the past year, how was your educational performance?” Educational performance is an important SEP indicator among adolescents, as it is crucial for each individual to obtain optimal educational qualifications, which lead to more prestigious employment possibilities [33, 34]. A Korean study found that educational achievement was an important mediator linking parental SEP to an adolescent’s future SEP [35]. In addition, single motherhood is an important mechanism for the reproduction of socioeconomic disadvantages [36].

### Statistical analysis

The SEP variables were presented as frequencies and percentages, and we used the chi-squared tests to compare distribution among these variables depending on multicultural status. We calculated the age-adjusted 12-month prevalence of depressive mood, suicidal ideation, suicidal plans, and suicide attempts using the direct standardization method with the 2010 Korean Census population being referent, after taking into account the primary sampling units, stratification, and sample weights of KYRBS. The effect of each SEP variable on the relationship between multicultural status and mental health was examined with prevalence ratios (PRs) and 95% confidence intervals (95% CIs), as determined by log-binomial regression with PROC GENMOD in the SAS statistical software (SAS Institute, Cary, NC, USA) [37]. If the binomial model failed to converge, the modified Poisson approach was used [37]. Separate analyses for males and females were conducted, and sample weights were applied to maintain the representativeness of the population. All statistical analyses were performed with SAS version 9.3.

## Results

Multicultural adolescents accounted for about 0.9% of the total sample of adolescents. Non-multicultural adolescents showed significantly higher positions regarding all SEP indicators considered than their multicultural counterparts (Table 1).

**Table 1:** Gender- and multicultural status-specific numbers and percentages of study subjects according to school grade and socioeconomic position indicators in the Korea Youth Risk Behavior Web-based Survey, 2011–2014

	Boys			Girls		
	Non-multicultural	Multi-cultural	*P*-value	Non-multicultural	Multi-cultural	*P*-value
	n (%)	n (%)		n (%)	n (%)	
Total	147848 (100.0)	1371 (100.0)		143809 (100.0)	1296 (100.0)	
School grades						
Middle school, 1st grade	25106 (17.0)	295 (21.5)	<.0001	23272 (16.2)	277 (21.4)	<.0001
Middle school, 2nd grade	25095 (17.0)	304 (22.2)		23982 (16.7)	294 (22.7)	
Middle school, 3rd grade	25253 (17.1)	264 (19.3)		24060 (16.7)	255 (19.7)	
High school, 1st grade	24935 (16.9)	203 (14.8)		23546 (16.4)	188 (14.5)	
High school, 2nd grade	23604 (16.0)	175 (12.8)		24900 (17.3)	161 (12.4)	
High school, 3rd grade	23855 (16.1)	130 (9.5)		24049 (16.7)	121 (9.3)	
Urbanity						
Metropolitan cities	67413 (45.6)	478 (34.9)	<.0001	64482 (44.8)	419 (32.3)	<.0001
Small and medium cities	63212 (42.8)	565 (41.2)		64149 (44.6)	556 (42.9)	
Rural areas	17223 (11.6)	328 (23.9)		15178 (10.6)	321 (24.8)	
Father’s education						
College	65876 (44.6)	319 (23.3)	<.0001	63406 (44.1)	276 (21.3)	<.0001
High school	46996 (31.8)	406 (29.6)		49667 (34.5)	411 (31.7)	
Middle school	5794 (3.9)	251 (18.3)		5578 (3.9)	240 (18.5)	
Do not know	22698 (15.4)	323 (23.6)		18997 (13.2)	286 (22.1)	
No father	6484 (4.4)	72 (5.3)		6161 (4.3)	83 (6.4)	
Mother’s education						
College	53414 (36.1)	390 (28.4)	<.0001	50462 (35.1)	429 (33.1)	<.0001
High school	58645 (39.7)	408 (29.8)		65331 (45.4)	443 (34.2)	
Middle school	5361 (3.6)	133 (9.7)		5735 (4.0)	114 (8.8)	
Do not know	23640 (16.0)	379 (27.6)		17033 (11.8)	275 (21.2)	
No mother	6788 (4.6)	61 (4.4)		5248 (3.6)	34 (2.6)	
Perceived household economic status						
High	13063 (8.8)	148 (10.8)	<.0001	7105 (4.9)	78 (6.0)	<.0001
Mid-high	37644 (25.5)	201 (14.7)		32990 (22.9)	176 (13.6)	
Middle	66836 (45.2)	561 (40.9)		72186 (50.2)	597 (46.1)	
Mid-low	23086 (15.6)	309 (22.5)		25202 (17.5)	324 (25.0)	
Low	7219 (4.9)	152 (11.1)		6326 (4.4)	121 (9.3)	
Self-reported educational performance						
High	18517 (12.5)	183 (13.3)	<.0001	14209 (9.9)	115 (8.9)	<.0001
Mid-high	34669 (23.4)	257 (18.7)		35625 (24.8)	231 (17.8)	
Middle	39468 (26.7)	317 (23.1)		40410 (28.1)	344 (26.5)	
Mid-low	35978 (24.3)	366 (26.7)		36964 (25.7)	379 (29.2)	
Low	19216 (13.0)	248 (18.1)		16601 (11.5)	227 (17.5)	
Cohabitation with parents						
Lives with both parents	122454 (82.8)	1000 (72.9)	<.0001	119063 (82.8)	996 (76.9)	<.0001
Lives with only mother	12817 (8.7)	151 (11.0)		14064 (9.8)	161 (12.4)	
Lives with only father	7543 (5.1)	101 (7.4)		6338 (4.4)	68 (5.2)	
Lives with no parents	5034 (3.4)	119 (8.7)		4344 (3.0)	71 (5.5)	

When we examined age-standardized 12-month prevalence rates of mental health outcomes that were unadjusted for SEP indicators, girls showed a relatively greater prevalence of poor mental health than boys, especially among non-multicultural adolescents. We also explored the gender differences in the risk of poor mental health and found significant results for all outcome measures (all *p* values <0.001). Multicultural boys reported a significantly greater prevalence of suicidal ideation, suicidal plans, and suicide attempts than non-multicultural boys. Boys and girls with two foreign-born parents generally had a greater prevalence for all four indicators of poor mental health than their non-multicultural counterparts. However, both boys and girls with Japan-born mothers had a lower prevalence of suicide plans than non-multicultural adolescents. Moreover, we were not able to find any significant differences between adolescents with Korean-Chinese mothers and non-multicultural adolescents regarding the prevalence of depressive mood and suicidality (Table 2).

**Table 2:** Age-standardized prevalence rates (95% confidence intervals) of mental health outcomes among Korean boys and girls according to multicultural status and parental country of birth: findings from the Korea Youth Risk Behavior Web-based Survey, 2011–2014

	Non-multicultural (both parents born in Korea)	Multicultural (at least one foreign-born parent)	Parental country of birth				
			Japan-born mother	Korean-Chinese mother	Other foreign-born mother	Foreign-born father	Both parents foreign-born
Boys							
n	147848	1371	345	258	364	172	232
Depressive mood	25.2 (24.9–25.5)	28.0 (25.1–30.9)	21.3 (16.4–26.1)	25.1 (19.0–31.1)	27.3 (21.8–32.9)	41.1 (32.1–50.2)	33.5 (25.7–41.4)
Suicidal ideation	13.2 (13.0–13.5)	17.4 (14.9–20.0)	9.9 (6.5–13.2)	12.3 (7.8–16.7)	17.5 (12.6–22.5)	26.6 (18.1–35.2)	29.6 (21.3–37.9)
Suicidal plans	4.7 (4.6–4.9)	8.2 (6.3–10.1)	1.6 (0.4–2.7)	3.1 (0.7–5.4)	9.3 (5.5–13.1)	16.3 (9.2–23.4)	18.0 (11.0–25.0)
Suicide attempt	2.6 (2.5–2.7)	6.7 (5.1–8.3)	2.2 (0.5–3.8)	1.6 (0.0–3.4)	7.8 (4.4–11.1)	13.3 (7.1–19.5)	14.0 (7.6–20.3)
Girls							
n	143809	1296	324	333	351	148	139
Depressive mood	35.8 (35.4–36.1)	37.4 (34.1–40.6)	27.9 (22.4–33.4)	36.0 (29.1–43.0)	40.0 (33.8–46.2)	45.9 (36.4–55.5)	48.4 (37.6–59.2)
Suicidal ideation	20.6 (20.3–20.9)	20.2 (17.6–22.9)	11.9 (7.7–16.1)	21.6 (16.4–26.9)	22.8 (17.6–28.1)	21.8 (13.9–29.7)	28.9 (17.9–40.0)
Suicidal plans	6.6 (6.5–6.8)	7.7 (5.9–9.5)	2.9 (0.4–5.5)	7.5 (3.9–11.1)	6.9 (3.9–9.9)	10.4 (5.0–15.7)	20.4 (10.8–30.0)
Suicide attempt	4.9 (4.7–5.0)	6.3 (4.7–8.0)	2.7 (0.5–5.0)	7.0 (3.5–10.4)	5.2 (2.4–7.9)	9.1 (3.7–14.4)	14.1 (5.4–22.8)

As shown in Table 3, the relationships between multicultural status and all four adverse mental health indicators were significant in the baseline model (adjusted only for age) among boys, whereas among girls, a significant relationship was only found for suicide attempts. When we examined the interactions between gender and multicultural status (multicultural vs. non-multicultural) for each of the outcome variables were considered in this study, we found generally significant gender differences (*p*-values <0.001) except for the interaction between gender and multicultural status for depressive mood (*p*-value = 0.0503). Among boys, the magnitude of the relationships between multicultural status and poor mental health was attenuated with adjustment of SEP indicators. In particular, a considerable reduction in the PRs was observed after adjusting for perceived household economic status. Nonetheless, even after adjusting for all six SEP indicators, the relationships of multicultural status with suicidal ideation, suicidal plans, and suicide attempts remained significant among boys. In girls, the baseline model suggested one significant association, between multicultural status and suicide attempts, but this association disappeared after adjustment for SEP indicators (Table 3).

**Table 3:** Prevalence ratios (95% confidence intervals) of poor mental health among Korean boys and girls by multicultural status (reference = non-multicultural status), after adjusting for age and socioeconomic position indicators: findings from the Korea Youth Risk Behavior Web-based Survey, 2011–2014

	Boys	Girls
	PR (95% CI)	PR (95% CI)
Depressive mood		
Baseline model (adjusted for age)	1.12 (1.02–1.24)	1.05 (0.96–1.14)
+ Urbanity	1.12 (1.02–1.23)	1.04 (0.96–1.13)
+ Father’s education	1.12 (1.01–1.23)	1.02 (0.94–1.11)
+ Mother’s education	1.13 (1.03–1.25)	1.05 (0.96–1.14)
+ Perceived household economic status	1.07 (0.97–1.17)	0.99 (0.92–1.08)
+ Self-reported educational performance	1.10 (1.00–1.21)	1.00 (0.92–1.09)
+ Cohabitation with parents	1.10 (1.00–1.21)	1.03 (0.95–1.12)
+ All socioeconomic position indicators	1.07 (0.97–1.18)	0.98 (0.90–1.06)
Suicidal ideation		
Baseline model (adjusted for age)	1.33 (1.16–1.51)	0.98 (0.87–1.11)
+ Urbanity	1.33 (1.17–1.51)	0.98 (0.87–1.11)
+ Father’s education	1.31 (1.15–1.49)	0.94 (0.83–1.06)
+ Mother’s education	1.33 (1.17–1.52)	0.98 (0.87–1.11)
+ Perceived household economic status	1.21 (1.06–1.37)	0.91 (0.81–1.03)
+ Self-reported educational performance	1.28 (1.13–1.46)	0.94 (0.83–1.06)
+ Cohabitation with parents	1.29 (1.13–1.47)	0.96 (0.85–1.08)
+ All socioeconomic position indicators	1.21 (1.06–1.37)	0.90 (0.80–1.01)
Suicidal plans		
Baseline model (adjusted for age)	1.74 (1.43–2.12)	1.16 (0.94–1.42)
+ Urbanity	1.73 (1.42–2.11)	1.16 (0.94–1.42)
+ Father’s education	1.69 (1.39–2.06)	1.08 (0.88–1.34)
+ Mother’s education	1.75 (1.43–2.13)	1.16 (0.94–1.43)
+ Perceived household economic status	1.57 (1.29–1.91)	1.05 (0.86–1.30)
+ Self-reported educational performance	1.67 (1.37–2.03)	1.08 (0.88–1.33)
+ Cohabitation with parents	1.65 (1.35–2.01)	1.10 (0.89–1.35)
+ All socioeconomic position indicators	1.52 (1.23–1.88)	1.03 (0.82–1.30)
Suicide attempts		
Baseline model (adjusted for age)	2.60 (2.08–3.24)	1.28 (1.02–1.61)
+ Urbanity	2.56 (2.05–3.20)	1.28 (1.01–1.61)
+ Father’s education	2.45 (1.96–3.06)	1.17 (0.93–1.47)
+ Mother’s education	2.60 (2.08–3.25)	1.28 (1.02–1.62)
+ Perceived household economic status	2.26 (1.81–2.81)	1.15 (0.91–1.45)
+ Self-reported educational performance	2.43 (1.95–3.04)	1.17 (0.93–1.47)
+ Cohabitation with parents	2.41 (1.93–3.00)	1.21 (0.96–1.52)
+ All socioeconomic position indicators	2.11 (1.67–2.68)	1.11 (0.86–1.44)

*PR* prevalence ratio, *CI* confidence interval

Tables 4 and 5 present the PRs of poor mental health according to parental country of birth. The baseline model showed that boys with foreign-born fathers and boys with two foreign-born parents were at a significantly greater risk of poor mental health than non-multicultural boys. The risk of depressive mood and suicidality among boys with foreign-born fathers and the risk of suicidality among boys with two foreign-born parents remained significant after adjustment of all six SEP indicators (Table 4). Meanwhile, in the baseline model, girls with foreign-born fathers had a significantly greater risk of depressive mood and suicide attempts, but the associations disappeared after adjustment of SEP indicators. While girls with two foreign-born parents had a significantly greater risk of depressive mood, suicidal plans, and suicide attempts in comparison with non-multicultural girls in the baseline model, and these associations remained significant after adjustment of SEP indicators (Table 5). The PRs in the baseline model for the three suicide-related indicators were significantly greater among boys with other foreign-born mothers than among their non-multicultural counterparts, and the association with suicidal plans and attempts remained significant after adjustment for all six SEP indicators (Table 4). The PRs for all four mental health indicators were not significantly elevated among adolescents with Korean-Chinese mothers. Compared to non-multicultural boys, boys with Japan-born mothers had a significantly lower PR of suicidal plans. Girls with Japan-born mothers had significantly lower PRs of depressive mood and suicidal ideation than non-multicultural girls. Tables 4 and 5 also demonstrate that adjustment of SEP indicators, especially perceived household economic status, resulted in a reduction in the PRs of poor mental health.

**Table 4:** Prevalence ratios (95% confidence intervals) of poor mental health among Korean boys according to parental country of birth (reference = both parents born in Korea), after adjusting for age and socioeconomic position indicators: findings from the Korea Youth Risk Behavior Web-based Survey, 2011–2014

	Japan-born mother	Korean-Chinese mother	Other foreign-born mother	Foreign-born father	Both parents foreign-born
	PR (95% CI)	PR (95% CI)	PR (95% CI)	PR (95% CI)	PR (95% CI)
Depressive mood					
Baseline model (adjusted for age)	0.84 (0.66–1.07)	1.00 (0.78–1.28)	1.09 (0.88–1.34)	1.62 (1.31–2.01)	1.32 (1.04–1.67)
+ Urbanity	0.84 (0.66–1.06)	1.00 (0.78–1.27)	1.09 (0.88–1.34)	1.62 (1.31–2.02)	1.32 (1.04–1.67)
+ Father’s education	0.83 (0.65–1.06)	0.97 (0.76–1.24)	1.09 (0.88–1.34)	1.65 (1.33–2.04)	1.32 (1.04–1.67)
+ Mother’s education	0.85 (0.66–1.08)	1.02 (0.79–1.30)	1.12 (0.91–1.38)	1.52 (1.23–1.89)	1.33 (1.05–1.69)
+ Perceived household economic status	0.81 (0.64–1.03)	0.94 (0.73–1.20)	1.04 (0.84–1.28)	1.49 (1.21–1.83)	1.22 (0.97–1.53)
+ Self-reported educational performance	0.83 (0.65–1.06)	0.96 (0.75–1.23)	1.04 (0.85–1.28)	1.57 (1.28–1.94)	1.27 (1.00–1.60)
+ Cohabitation with parents	0.85 (0.66–1.08)	0.98 (0.77–1.25)	1.07 (0.87–1.32)	1.51 (1.22–1.87)	1.27 (1.00–1.60)
+ All socioeconomic position indicators	0.82 (0.65–1.05)	0.95 (0.74–1.21)	1.05 (0.85–1.29)	1.41 (1.16–1.73)	1.19 (0.95–1.49)
Suicidal ideation					
Baseline model (adjusted for age)	0.74 (0.52–1.05)	0.93 (0.64–1.35)	1.33 (1.01–1.76)	2.01 (1.47–2.75)	2.22 (1.70–2.90)
+ Urbanity	0.74 (0.53–1.05)	0.93 (0.64–1.35)	1.33 (1.01–1.76)	2.01 (1.47–2.75)	2.22 (1.70–2.90)
+ Father’s education	0.74 (0.52–1.04)	0.90 (0.62–1.31)	1.32 (1.00–1.74)	2.03 (1.49–2.77)	2.21 (1.69–2.88)
+ Mother’s education	0.75 (0.53–1.07)	0.95 (0.65–1.38)	1.37 (1.04–1.81)	1.79 (1.32–2.43)	2.21 (1.70–2.88)
+ Perceived household economic status	0.71 (0.50–1.00)	0.85 (0.58–1.23)	1.25 (0.94–1.65)	1.73 (1.27–2.36)	1.93 (1.48–2.51)
+ Self-reported educational performance	0.74 (0.53–1.05)	0.90 (0.62–1.31)	1.27 (0.96–1.68)	1.91 (1.40–2.59)	2.08 (1.59–2.71)
+ Cohabitation with parents	0.75 (0.53–1.07)	0.91 (0.63–1.32)	1.30 (0.98–1.72)	1.80 (1.32–2.45)	2.07 (1.59–2.68)
+ All socioeconomic position indicators	0.73 (0.52–1.04)	0.88 (0.60–1.28)	1.27 (0.96–1.68)	1.57 (1.16–2.13)	1.82 (1.39–2.36)
Suicidal plans					
Baseline model (adjusted for age)	0.34 (0.16–0.70)	0.66 (0.32–1.38)	1.95 (1.32–2.88)	3.47 (2.24–5.39)	3.86 (2.63–5.66)
+ Urbanity	0.33 (0.16–0.69)	0.66 (0.31–1.37)	1.94 (1.31–2.86)	3.47 (2.23–5.38)	3.85 (2.62–5.65)
+ Father’s education	0.33 (0.16–0.69)	0.62 (0.29–1.29)	1.89 (1.27–2.79)	3.51 (2.26–5.44)	3.78 (2.60–5.51)
+ Mother’s education	0.35 (0.17–0.72)	0.70 (0.33–1.45)	2.05 (1.39–3.03)	2.74 (1.79–4.21)	3.88 (2.67–5.65)
+ Perceived household economic status	0.33 (0.16–0.69)	0.61 (0.29–1.28)	1.82 (1.24–2.68)	2.69 (1.74–4.17)	2.96 (2.06–4.24)
+ Self-reported educational performance	0.34 (0.17–0.71)	0.64 (0.31–1.34)	1.84 (1.25–2.71)	3.18 (2.06–4.92)	3.44 (2.36–5.01)
+ Cohabitation with parents	0.35 (0.17–0.72)	0.64 (0.31–1.34)	1.86 (1.26–2.76)	2.91 (1.88–4.49)	3.37 (2.31–4.93)
+ All socioeconomic position indicators	0.34 (0.16–0.70)	0.64 (0.30–1.33)	1.86 (1.27–2.72)	2.27 (1.49–3.47)	2.78 (1.95–3.97)
Suicide attempts					
Baseline model (adjusted for age)	0.84 (0.39–1.82)	0.65 (0.22–1.88)	2.97 (1.92–4.59)	5.16 (3.23–8.26)	5.45 (3.49–8.52)
+ Urbanity	0.82 (0.38–1.77)	0.64 (0.22–1.85)	2.92 (1.89–4.52)	5.16 (3.22–8.25)	5.45 (3.48–8.51)
+ Father’s education	0.81 (0.37–1.76)	0.57 (0.19–1.66)	2.75 (1.77–4.26)	5.26 (3.30–8.38)	5.26 (3.40–8.12)
+ Mother’s education	0.87 (0.40–1.89)	0.68 (0.23–1.99)	3.10 (2.00–4.79)	3.90 (2.51–6.05)	5.60 (3.60–8.71)
+ Perceived household economic status	0.82 (0.38–1.79)	0.59 (0.20–1.73)	2.73 (1.76–4.23)	3.73 (2.36–5.88)	3.87 (2.55–5.87)
+ Self-reported educational performance	0.85 (0.39–1.85)	0.63 (0.21–1.83)	2.73 (1.77–4.23)	4.52 (2.85–7.17)	4.58 (2.97–7.07)
+ Cohabitation with parents	0.88 (0.40–1.90)	0.62 (0.21–1.80)	2.78 (1.79–4.33)	4.02 (2.55–6.34)	4.49 (2.93–6.89)
+ All socioeconomic position indicators	0.83 (0.38–1.81)	0.58 (0.20–1.71)	2.64 (1.70–4.11)	3.15 (2.06–4.80)	3.47 (2.32–5.19)

*PR* prevalence ratio, *CI* confidence interval

**Table 5:** Prevalence ratios (95% confidence intervals) of poor mental health among Korean girls according to parental country of birth (reference = both parents born in Korea), after adjusting for age and socioeconomic position indicators: findings from the Korea Youth Risk Behavior Web-based Survey, 2011–2014

	Japan-born mother	Korean Chinese mother	Other foreign-born mother	Foreign-born father	Both parents foreign-born
	PR (95% CI)	PR (95% CI)	PR (95% CI)	PR (95% CI)	PR (95% CI)
Depressive mood					
Baseline model (adjusted for age)	0.77 (0.63–0.95)	1.01 (0.83–1.23)	1.12 (0.96–1.32)	1.29 (1.04–1.58)	1.35 (1.08–1.69)
+ Urbanity	0.77 (0.62–0.94)	1.00 (0.82–1.22)	1.11 (0.95–1.31)	1.29 (1.05–1.58)	1.35 (1.08–1.69)
+ Father’s education	0.77 (0.62–0.94)	0.97 (0.80–1.17)	1.08 (0.92–1.27)	1.30 (1.06–1.60)	1.34 (1.07–1.68)
+ Mother’s education	0.79 (0.64–0.97)	1.01 (0.82–1.23)	1.14 (0.97–1.33)	1.25 (1.02–1.55)	1.34 (1.07–1.67)
+ Perceived household economic status	0.74 (0.60–0.91)	0.97 (0.79–1.18)	1.08 (0.92–1.26)	1.22 (0.99–1.50)	1.29 (1.03–1.62)
+ Self-reported educational performance	0.77 (0.62–0.94)	0.96 (0.78–1.18)	1.06 (0.90–1.24)	1.22 (1.00–1.49)	1.33 (1.05–1.68)
+ Cohabitation with parents	0.79 (0.64–0.97)	0.99 (0.81–1.20)	1.10 (0.94–1.29)	1.22 (0.99–1.51)	1.26 (1.01–1.57)
+ All socioeconomic position indicators	0.74 (0.61–0.91)	0.96 (0.78–1.18)	1.03 (0.88–1.21)	1.14 (0.93–1.40)	1.27 (1.00–1.61)
Suicidal ideation					
Baseline model (adjusted for age)	0.59 (0.41–0.83)	1.05 (0.82–1.34)	1.11 (0.88–1.39)	1.06 (0.74–1.53)	1.41 (0.98–2.03)
+ Urbanity	0.59 (0.41–0.83)	1.05 (0.82–1.34)	1.11 (0.88–1.39)	1.06 (0.74–1.53)	1.41 (0.98–2.03)
+ Father’s education	0.57 (0.40–0.81)	0.98 (0.76–1.25)	1.03 (0.82–1.29)	1.08 (0.75–1.55)	1.38 (0.96–1.96)
+ Mother’s education	0.60 (0.42–0.85)	1.04 (0.82–1.33)	1.12 (0.89–1.41)	1.03 (0.71–1.49)	1.38 (0.96–1.98)
+ Perceived household economic status	0.54 (0.38–0.77)	0.98 (0.77–1.25)	1.03 (0.82–1.29)	0.97 (0.67–1.40)	1.30 (0.92–1.84)
+ Self-reported educational performance	0.58 (0.41–0.83)	0.99 (0.78–1.26)	1.04 (0.83–1.30)	1.00 (0.69–1.44)	1.37 (0.95–1.97)
+ Cohabitation with parents	0.60 (0.43–0.86)	1.02 (0.80–1.30)	1.07 (0.85–1.34)	0.98 (0.68–1.42)	1.27 (0.89–1.82)
+ All socioeconomic position indicators	0.56 (0.40–0.79)	0.97 (0.76–1.23)	0.99 (0.79–1.24)	0.90 (0.63–1.29)	1.23 (0.87–1.75)
Suicidal plans					
Baseline model (adjusted for age)	0.48 (0.22–1.03)	1.12 (0.70–1.80)	1.03 (0.69–1.54)	1.57 (0.94–2.63)	3.20 (1.99–5.15)
+ Urbanity	0.48 (0.22–1.03)	1.12 (0.70–1.80)	1.03 (0.69–1.54)	1.57 (0.94–2.63)	3.20 (1.98–5.15)
+ Father’s education	0.47 (0.22–1.01)	1.01 (0.63–1.62)	0.94 (0.62–1.40)	1.61 (0.97–2.69)	3.11 (1.95–4.95)
+ Mother’s education	0.49 (0.23–1.07)	1.12 (0.70–1.79)	1.05 (0.70–1.57)	1.47 (0.88–2.47)	3.09 (1.93–4.93)
+ Perceived household economic status	0.44 (0.20–0.95)	1.04 (0.65–1.66)	0.95 (0.64–1.42)	1.38 (0.83–2.31)	2.70 (1.70–4.28)
+ Self-reported educational performance	0.48 (0.22–1.04)	1.04 (0.65–1.66)	0.95 (0.64–1.42)	1.45 (0.86–2.43)	3.05 (1.86–4.98)
+ Cohabitation with parents	0.50 (0.23–1.08)	1.07 (0.67–1.72)	0.97 (0.65–1.46)	1.38 (0.83–2.30)	2.64 (1.66–4.20)
+ All socioeconomic position indicators	0.47 (0.22–1.02)	1.03 (0.64–1.65)	0.90 (0.60–1.35)	1.24 (0.74–2.08)	2.48 (1.54–3.99)
Suicide attempt					
Baseline model (adjusted for age)	0.60 (0.29–1.22)	1.40 (0.87–2.26)	1.05 (0.65–1.68)	1.88 (1.05–3.38)	3.01 (1.61–5.63)
+ Urbanity	0.60 (0.29–1.22)	1.39 (0.86–2.25)	1.04 (0.65–1.67)	1.89 (1.05–3.39)	3.01 (1.61–5.63)
+ Father’s education	0.57 (0.28–1.17)	1.22 (0.76–1.96)	0.92 (0.57–1.48)	1.95 (1.09–3.50)	2.85 (1.55–5.24)
+ Mother’s education	0.63 (0.31–1.28)	1.39 (0.86–2.24)	1.07 (0.67–1.71)	1.73 (0.96–3.11)	2.90 (1.57–5.38)
+ Perceived household economic status	0.54 (0.26–1.11)	1.28 (0.79–2.06)	0.96 (0.60–1.54)	1.64 (0.92–2.92)	2.51 (1.36–4.63)
+ Self-reported educational performance	0.59 (0.29–1.21)	1.26 (0.78–2.04)	0.94 (0.58–1.50)	1.68 (0.94–2.99)	2.84 (1.49–5.39)
+ Cohabitation with parents	0.63 (0.31–1.30)	1.32 (0.82–2.13)	0.98 (0.61–1.58)	1.63 (0.90–2.93)	2.42 (1.32–4.43)
+ All socioeconomic position indicators	0.58 (0.28–1.20)	1.25 (0.78–2.01)	0.87 (0.54–1.40)	1.44 (0.81–2.57)	2.31 (1.25–4.25)

*PR* prevalence ratio, *CI* confidence interval

## Discussion

The results of this study showed significant differences between multicultural and non-multicultural boys regarding the prevalence of suicidality (suicide ideation, suicide plans, and suicide attempts). The impact of multicultural status on mental health varied depending on the foreign-born status of the parents and the maternal country of birth. Compared with non-multicultural counterparts, boys with Japan-born mothers showed lower PRs of suicidal plans and girls with Japan-born mothers showed lower PRs of depressive mood and suicidal ideation. While, adolescents with Korean-Chinese mothers showed similar PRs of depressed mood and suicidality compared with non-multicultural adolescents. However, boys with foreign-born fathers and boys with two foreign-born parents showed greater PRs of suicidality, and girls with two foreign-born parents showed greater PRs of suicidal plans and suicide attempts. In addition, the magnitude of the relationship between multicultural status and mental health was attenuated with adjustment of SEP indicators. Perceived household economic status played a particularly important role in this attenuation.

In this study, SEP indicators might have played a role as mediators in the relationship between multicultural status and mental health. The differences in outcome measures after adjustment of SEP indicators might show the disadvantages in depressive mood and suicidality which were not attributable to socioeconomic situations that multicultural adolescents face. Prior studies have identified that the risk factors for poor mental health among the children of immigrants include poor parenting and family functioning, low language competence, issues relating to cultural identity, discrimination, and low SEP [2, 4, 6, 9, 14, 19]. The results of our analysis, in which different PRs of poor mental health were found depending on paternal foreign-born status and maternal country of birth, might be partly explained by different experiences of discrimination among different multicultural subgroups. Adolescents with Korean-Chinese mothers showed similar PRs of mental health outcomes compared with non-multicultural adolescents, which might be because Korean-Chinese adolescents can learn Korean easily and are similar in appearance to non-multicultural adolescents. Korean-Chinese adolescents might have experienced less discrimination and fewer identity issues. For example, according to the Longitudinal Study of Children and Adolescents from Multicultural Families in Korea, 85% of children whose mother was Korean-Chinese identified themselves as Korean and 13% of them identified as both Korean and Korean-Chinese [38].

The decreased age-standardized prevalence of depressed mood and suicidality found among adolescents with Japan-born mothers might be attributable to the high education levels of their mothers and to the presence of stable family structures (see Additional file 1: Tables S2 and S3). In addition to these factors, adolescents with Japan-born mothers might have been less discriminated against than other multicultural subgroups, due to both their physical appearance, which is similar to that of adolescents with Korean-born parents, and the wealth of their mothers’ home country. A Spanish study showed that perceived discrimination was higher among immigrants from low income countries than immigrants from high income countries [25]. According to the Korean National Survey of Multicultural Families in 2012, the offspring of Japanese migrants reported the highest rate of positive identity associated with multicultural status among all offspring of Asian immigrants [39].

In our study, multicultural boys with foreign-born fathers and boys with two foreign-born parents showed greater PRs of suicidality than non-multicultural boys, and girls with two foreign-born parents showed greater PRs of suicidal plans and suicide attempts. Although boys with foreign-born fathers reported relatively high levels of paternal education, perceived household economic status, and self-reported educational performance, they showed the lowest rate of cohabitation with both parents among the subgroups analyzed in this study (see Additional file 1: Table S2). Children growing up with single parents were more likely to experience cognitive, emotional, and social problems [40]. Adolescents with two foreign-born parents also showed greater PRs of suicidal plans and suicide attempts, which might be partly due to their generally lower SEP, including lower education levels of both parents, low perceived household economic status, and low self-reported educational performance, as well as the relatively high proportion of these adolescents who lived with no parents (see Additional file 1: Tables S2 and S3).

The differential impacts of maternal versus paternal foreign-born status on mental health might be explained by the patriarchal and androcentric characteristics embedded in Korean society, which places a great emphasis on the paternal bloodline [41, 42]. It is possible that adolescent with foreign-born fathers might have been more alienated from their peers than adolescent with foreign-born mothers in the Korean society. Although KYRBS did not provide information about the adolescents’ country of birth, many adolescents with two foreign-born parents might also be foreign-born themselves. Multicultural children who grew up abroad experienced more discrimination than multicultural children who grew up in Korea [39]. In addition, it should be noted that these adolescents might have experienced linguistic inadequacy and cultural stress since both parents were foreign-born.

This study also found gender-related disparities. Boys with foreign-born mothers, with the exception of those whose mothers were born in Japan or were Korean-Chinese, showed greater PRs of suicidal plans and suicide attempts than non-multicultural boys, while girls showed no significant differences regarding those mental health indicators. In a Norwegian study, immigrant boys reported a greater prevalence of anxiety and depression than host country boys, while no significant differences were found among girls [43]. We assume that these gender differences might be due to greater levels of susceptibility among boys than girls to the negative mental health impacts of discrimination related to having a minority mother and having a different physical appearance, including skin color. Gender differences in strategies for coping with discrimination might also exist [14, 44]. When multicultural adolescents experienced discrimination, girls were more likely than boys to demand an apology and to discuss the situation with parents, teachers, and friends [39].

The results of our analysis showed that perceived household economic status was the SEP variable that played the most important role in the relationship between multicultural status and mental health. A previous Korean study reported a strong positive relationship between perceived household economic status and self-rated health [32]. Our results suggest that adolescents’ perceptions of a lack of material resources may be an important mechanism leading to an increased risk for poor mental health among some multicultural adolescents. Nonetheless, the associations of parental country of birth with suicidal plans and attempts remained significant after adjusting for SEP variables, especially among boys with a foreign-born father, boys with two foreign-born parents, and girls with two foreign-born parents. Mental health indicators among these subgroups might have been influenced by factors other than the SEP indicators considered in this study, such as discrimination, the presence of a language barrier, and issues relating to cultural identity. However, no variables reflecting these factors were available in the KYRBS data.

A major strength of this study is that, to the best of our knowledge, it is the first Korean study to examine adolescent mental health among multiple ethnic subgroups in comparison with non-multicultural adolescents. This was possible since we employed combined samples of several waves of KYRBS. Most previously published Korean studies, with one exception [10], dealing with these issues did not include a non-multicultural population in their study samples [11–14]. A previous Korean study examined differences in adolescent mental health between multicultural and non-multicultural adolescents, but was not able to examine subgroups within the population of multicultural adolescents [10].

This study also has some limitations. First, KYRBS did not contain information about each adolescent’s country of birth and period of residence in Korea, preventing us from adjusting for the length of stay in Korea. We also could not distinguish between Korea-born and foreign-born adolescents, and could not identify the immigrant generation to which each adolescent belonged. A prior study showed that second-generation immigrant youth were more likely to have suicidality than first-generation youth, especially among Hispanics and Asians [9]. According to the Korean National Survey of Multicultural Families in 2012, only 26.9% of children in multicultural families were seemingly the first-generation immigrants [39]. In this regard, the analysis results of our study might be the findings from second-generation immigrant adolescents. Second, the percentage of multicultural children enrolled in school is lower than the national average. Overall, 96.1% of Korean children attend middle school and 92.6% of children attend high school, whereas 92.3% of multicultural children attend middle school and 85.1% attend high school [39]. Since KYRBS is a school-based survey, it did not include adolescents who did not attend school or had dropped out of school. It is therefore possible that the relationships between mental health and multicultural status were underestimated. Third, KYRBS was a cross-sectional survey which cannot establish a temporal ordering of the associations, thus reverse causality may be implicated. For example, depressive mood may lead to reporting lower SEP.

## Conclusions

An increasing concern has emerged about mental health problems and suicidal behaviors among adolescents in Korea. The results of our study demonstrated differences in mental health according to parental foreign-born status, including maternal country of birth, and showed that the relationship between mental health and multicultural status was partially mediated by SEP. In order to ensure the effective implementation of policies to reduce mental health problems among multicultural adolescents in Korea, detailed information should be considered on the cultural and socioeconomic backgrounds of families, such as parental country of birth and SEP.

## Additional file

Additional file 1: Supplemental figure and tables﻿. (ZIP 134 kb)

## Abbreviations

CIs: confidence intervals
KYRBS: Korea Youth Risk Behavior Web-based Survey
PR: Prevalence ratio
SEP: Socioeconomic position
