# Supplementary material for: Associations of multicultural status with depressive mood and suicidality among Korean adolescents: the roles of parental country of birth and socioeconomic position
Source: BMC Public Health. 2017 Jan 25;17:116. doi: 10.1186/s12889-017-4044-y (PMC5264283; doi:10.1186/s12889-017-4044-y)
Supplement: Additional file 1: — Supplemental figure and tables﻿. (ZIP 134 kb) [file 12889_2017_4044_MOESM1_ESM.zip › Updated supplemental figure and table/Supplemental figure and tablesï»¿.docx]

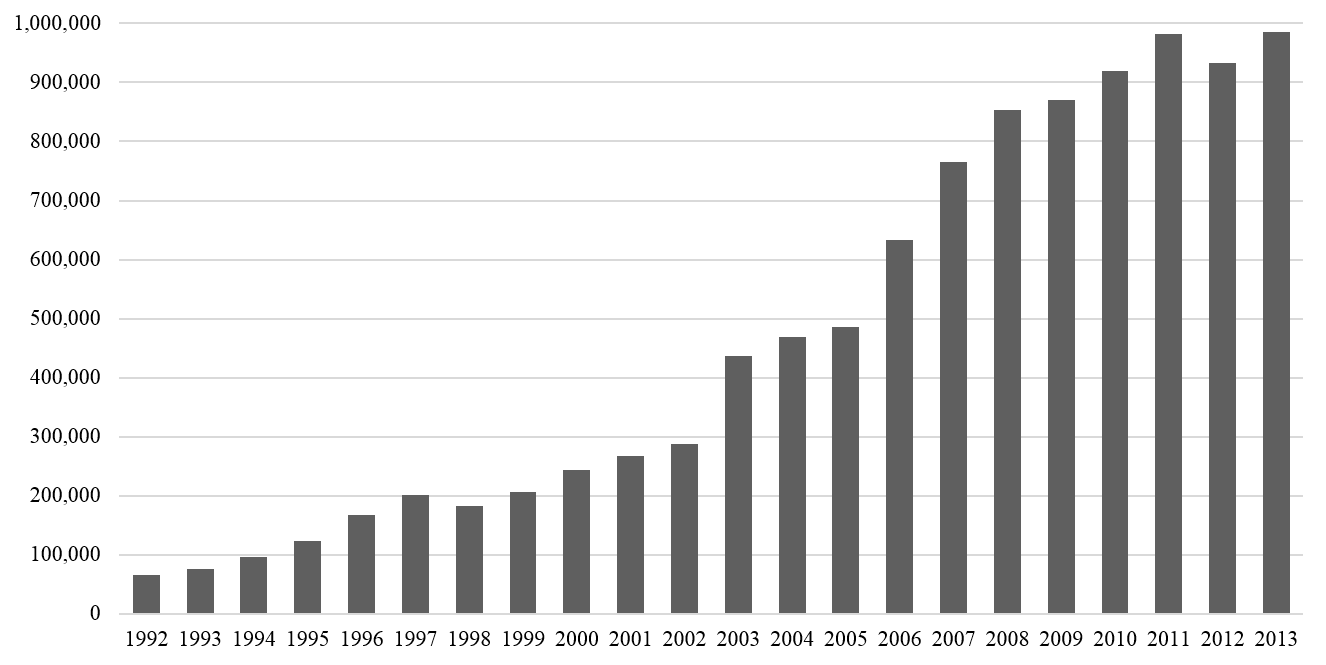


Figure S1. The registered foreign population in Korea, 1992–2013 (Data from Korean Statistical Information Service, 2015).

Table S1. Numbers and percentages of parental country of birth among adolescents with at least one foreign-born parent.

|  | Foreign-born mother | Foreign-born father | Both parents foreign-born | |
| --- | --- | --- | --- | --- |
|  | Mother's country of birth | Father's country of birth | Mother's country of birth | Father's country of birth |
| Korean-Chinese | 591 (29.9) | 30 (9.4) | 64 (17.3) | 74 (19.9) |
| China | 231 (11.7) | 35 (10.9) | 33 (8.9) | 23 (6.2) |
| North Korea | 50 (2.5) | 31 (9.7) | 82 (22.1) | 84 (22.6) |
| Vietnam | 76 (3.8) | 13 (4.1) | 24 (6.5) | 22 (5.9) |
| Philippines | 215 (10.9) | 11 (3.4) | 18 (4.9) | 17 (4.6) |
| Japan | 669 (33.9) | 90 (28.1) | 23 (6.2) | 24 (6.5) |
| Taiwan | 13 (0.7) | 11 (3.4) | 9 (2.4) | 11 (3) |
| Mongolia | 19 (1.0) | 5 (1.6) | 27 (7.3) | 30 (8.1) |
| Thailand | 27 (1.4) | 1 (0.3) | 6 (1.6) | 2 (0.5) |
| Cambodia | 11 (0.6) | 5 (1.6) | 14 (3.8) | 11 (3) |
| Uzbekistan | 6 (0.3) | 11 (3.4) | 20 (5.4) | 30 (8.1) |
| Russia | 12 (0.6) | 7 (2.2) | 17 (4.6) | 12 (3.2) |
| Other | 55 (2.8) | 70 (21.9) | 34 (9.2) | 31 (8.4) |
| Total | 1975 (100.0) | 320 (100.0) | 371 (100.0) | 371 (100) |

Table S2. Number and percentage of male study subjects by parental country of birth, according to school grades and socioeconomic position indicators: findings from the Youth Risk Behavior Web-based Survey, 2011–2014.

|  | Both parents born in Korea | Japan-born mother | Korean Chinese mother | Other foreign-born mother | Foreign-born father | Both parents foreign-born | P-value |
| --- | --- | --- | --- | --- | --- | --- | --- |
|  | n (%) | n (%) | n (%) | n (%) | n (%) | n (%) |  |
| School grades |  |  |  |  |  |  |  |
| Middle school, 1st grade | 25106 (17.0) | 69 (20.0) | 58 (22.5) | 112 (30.8) | 28 (16.3) | 28 (12.1) | <.0001 |
| Middle school, 2nd grade | 25095 (17.0) | 94 (27.2) | 57 (22.1) | 85 (23.4) | 27 (15.7) | 41 (17.7) |  |
| Middle school, 3rd grade | 25253 (17.1) | 66 (19.1) | 59 (22.9) | 52 (14.3) | 35 (20.3) | 52 (22.4) |  |
| High school, 1st grade | 24935 (16.9) | 50 (14.5) | 40 (15.5) | 54 (14.8) | 26 (15.1) | 33 (14.2) |  |
| High school, 2nd grade | 23604 (16.0) | 44 (12.8) | 32 (12.4) | 31 (8.5) | 27 (15.7) | 41 (17.7) |  |
| High school, 3rd grade | 23855 (16.1) | 22 (6.4) | 12 (4.7) | 30 (8.2) | 29 (16.9) | 37 (15.9) |  |
| Urbanity |  |  |  |  |  |  |  |
| Metropolitan cities | 67413 (45.6) | 83 (24.1) | 79 (30.6) | 117 (32.1) | 88 (51.2) | 111 (47.8) | <.0001 |
| Small and medium cities | 63212 (42.8) | 137 (39.7) | 114 (44.2) | 155 (42.6) | 64 (37.2) | 95 (40.9) |  |
| Rural areas | 17223 (11.6) | 125 (36.2) | 65 (25.2) | 92 (25.3) | 20 (11.6) | 26 (11.2) |  |
| Father’s education |  |  |  |  |  |  |  |
| College | 65876 (44.6) | 93 (27.0) | 20 (7.8) | 67 (18.4) | 70 (40.7) | 69 (29.7) | <.0001 |
| High school | 46996 (31.8) | 116 (33.6) | 111 (43.0) | 112 (30.8) | 31 (18.0) | 36 (15.5) |  |
| Middle school | 5794 (3.9) | 61 (17.7) | 58 (22.5) | 43 (11.8) | 29 (16.9) | 60 (25.9) |  |
| Do not know | 22698 (15.4) | 65 (18.8) | 45 (17.4) | 104 (28.6) | 42 (24.4) | 67 (28.9) |  |
| No father | 6484 (4.4) | 10 (2.9) | 24 (9.3) | 38 (10.4) | 0 (0.0) | 0 (0.0) |  |
| Mother’s education |  |  |  |  |  |  |  |
| College | 53414 (36.1) | 156 (45.2) | 43 (16.7) | 95 (26.1) | 44 (25.6) | 52 (22.4) | <.0001 |
| High school | 58645 (39.7) | 112 (32.5) | 101 (39.1) | 108 (29.7) | 34 (19.8) | 53 (22.8) |  |
| Middle school | 5361 (3.6) | 7 (2.0) | 29 (11.2) | 25 (6.9) | 11 (6.4) | 61 (26.3) |  |
| Do not know | 23640 (16.0) | 70 (20.3) | 85 (32.9) | 136 (37.4) | 22 (12.8) | 66 (28.4) |  |
| No mother | 6788 (4.6) | 0 (0.0) | 0 (0.0) | 0 (0.0) | 61 (35.5) | 0 (0.0) |  |
| Perceived household economic status |  |  |  |  |  |  |  |
| High | 13063 (8.8) | 12 (3.5) | 11 (4.3) | 26 (7.1) | 32 (18.6) | 67 (28.9) | <.0001 |
| Mid-high | 37644 (25.5) | 42 (12.2) | 39 (15.1) | 64 (17.6) | 29 (16.9) | 27 (11.6) |  |
| Middle | 66836 (45.2) | 173 (50.1) | 112 (43.4) | 160 (44.0) | 54 (31.4) | 62 (26.7) |  |
| Mid-low | 23086 (15.6) | 97 (28.1) | 73 (28.3) | 87 (23.9) | 23 (13.4) | 29 (12.5) |  |
| Low | 7219 (4.9) | 21 (6.1) | 23 (8.9) | 27 (7.4) | 34 (19.8) | 47 (20.3) |  |
| Self-reported educational performance |  |  |  |  |  |  |  |
| High | 18517 (12.5) | 28 (8.1) | 23 (8.9) | 30 (8.2) | 33 (19.2) | 69 (29.7) | <.0001 |
| Mid-high | 34669 (23.4) | 86 (24.9) | 48 (18.6) | 61 (16.8) | 36 (20.9) | 26 (11.2) |  |
| Middle | 39468 (26.7) | 101 (29.3) | 67 (26.0) | 90 (24.7) | 24 (14.0) | 35 (15.1) |  |
| Mid-low | 35978 (24.3) | 95 (27.5) | 79 (30.6) | 118 (32.4) | 34 (19.8) | 40 (17.2) |  |
| Low | 19216 (13.0) | 35 (10.1) | 41 (15.9) | 65 (17.9) | 45 (26.2) | 62 (26.7) |  |
| Cohabitation with parents |  |  |  |  |  |  |  |
| Lives with both parents | 122454 (82.8) | 310 (89.9) | 197 (76.4) | 271 (74.5) | 72 (41.9) | 150 (64.7) | <.0001 |
| Lives with only mother | 12817 (8.7) | 30 (8.7) | 31 (12.0) | 42 (11.5) | 26 (15.1) | 22 (9.5) |  |
| Lives with only father | 7543 (5.1) | 3 (0.9) | 18 (7.0) | 31 (8.5) | 36 (20.9) | 13 (5.6) |  |
| Lives with no parents | 5034 (3.4) | 2 (0.6) | 12 (4.7) | 20 (5.5) | 38 (22.1) | 47 (20.3) |  |

Table S3. Number and percentage of female study subjects by parental country of birth, according to school grades and socioeconomic position indicators: findings from the Korea Youth Risk Behavior Web-based Survey, 2011–2014.

|  | Both parents born in Korea | Japan-born mother | Korean Chinese mother | Other foreign-born mother | Foreign-born father | Both parents foreign-born | P-value |
| --- | --- | --- | --- | --- | --- | --- | --- |
|  | n (%) | n (%) | n (%) | n (%) | n (%) | n (%) |  |
| School grades |  |  |  |  |  |  |  |
| Middle school, 1st grade | 23272 (16.2) | 66 (20.4) | 69 (20.7) | 108 (30.8) | 16 (10.8) | 18 (12.9) | <.0001 |
| Middle school, 2nd grade | 23982 (16.7) | 81 (25.0) | 83 (24.9) | 78 (22.2) | 29 (19.6) | 23 (16.5) |  |
| Middle school, 3rd grade | 24060 (16.7) | 58 (17.9) | 73 (21.9) | 70 (19.9) | 29 (19.6) | 25 (18.0) |  |
| High school, 1st grade | 23546 (16.4) | 46 (14.2) | 51 (15.3) | 40 (11.4) | 23 (15.5) | 27 (19.4) |  |
| High school, 2nd grade | 24900 (17.3) | 44 (13.6) | 43 (12.9) | 30 (8.5) | 29 (19.6) | 15 (10.8) |  |
| High school, 3rd grade | 24049 (16.7) | 29 (9.0) | 14 (4.2) | 25 (7.1) | 22 (14.9) | 31 (22.3) |  |
| Urbanity |  |  |  |  |  |  |  |
| Metropolitan cities | 64482 (44.8) | 103 (31.8) | 94 (28.2) | 91 (25.9) | 75 (50.7) | 55 (39.6) | <.0001 |
| Small and medium cities | 64149 (44.6) | 109 (33.6) | 150 (45.0) | 162 (46.2) | 63 (42.6) | 72 (51.8) |  |
| Rural areas | 15178 (10.6) | 112 (34.6) | 89 (26.7) | 98 (27.9) | 10 (6.8) | 12 (8.6) |  |
| Father’s education |  |  |  |  |  |  |  |
| College | 63406 (44.1) | 92 (28.4) | 27 (8.1) | 49 (14.0) | 78 (52.7) | 29 (20.9) | <.0001 |
| High school | 49667 (34.5) | 122 (37.7) | 128 (38.4) | 104 (29.6) | 25 (16.9) | 32 (23.0) |  |
| Middle school | 5578 (3.9) | 53 (16.4) | 61 (18.3) | 75 (21.4) | 20 (13.5) | 31 (22.3) |  |
| Do not know | 18997 (13.2) | 49 (15.1) | 84 (25.2) | 81 (23.1) | 25 (16.9) | 47 (33.8) |  |
| No father | 6161 (4.3) | 8 (2.5) | 33 (9.9) | 42 (12.0) | 0 (0.0) | 0 (0.0) |  |
| Mother’s education |  |  |  |  |  |  |  |
| College | 50462 (35.1) | 159 (49.1) | 58 (17.4) | 128 (36.5) | 50 (33.8) | 34 (24.5) | <.0001 |
| High school | 65331 (45.4) | 130 (40.1) | 138 (41.4) | 96 (27.4) | 40 (27.0) | 39 (28.1) |  |
| Middle school | 5735 (4.0) | 1 (0.3) | 48 (14.4) | 24 (6.8) | 8 (5.4) | 33 (23.7) |  |
| Do not know | 17033 (11.8) | 34 (10.5) | 89 (26.7) | 103 (29.3) | 16 (10.8) | 33 (23.7) |  |
| No mother | 5248 (3.6) | 0 (0.0) | 0 (0.0) | 0 (0.0) | 34 (23.0) | 0 (0.0) |  |
| Perceived household economic status |  |  |  |  |  |  |  |
| High | 7105 (4.9) | 7 (2.2) | 8 (2.4) | 17 (4.8) | 16 (10.8) | 30 (21.6) | <.0001 |
| Mid-high | 32990 (22.9) | 31 (9.6) | 38 (11.4) | 54 (15.4) | 30 (20.3) | 22 (15.8) |  |
| Middle | 72186 (50.2) | 168 (51.9) | 180 (54.1) | 170 (48.4) | 42 (28.4) | 37 (26.6) |  |
| Mid-low | 25202 (17.5) | 94 (29.0) | 87 (26.1) | 85 (24.2) | 41 (27.7) | 17 (12.2) |  |
| Low | 6326 (4.4) | 24 (7.4) | 20 (6.0) | 25 (7.1) | 19 (12.8) | 33 (23.7) |  |
| Self-reported educational performance |  |  |  |  |  |  |  |
| High | 14209 (9.9) | 20 (6.2) | 26 (7.8) | 23 (6.6) | 18 (12.2) | 28 (20.1) | <.0001 |
| Mid-high | 35625 (24.8) | 73 (22.5) | 76 (22.8) | 46 (13.1) | 20 (13.5) | 16 (11.5) |  |
| Middle | 40410 (28.1) | 109 (33.6) | 74 (22.2) | 102 (29.1) | 30 (20.3) | 29 (20.9) |  |
| Mid-low | 36964 (25.7) | 88 (27.2) | 97 (29.1) | 113 (32.2) | 46 (31.1) | 34 (24.5) |  |
| Low | 16601 (11.5) | 34 (10.5) | 60 (18.0) | 67 (19.1) | 34 (23.0) | 32 (23.0) |  |
| Cohabitation with parents |  |  |  |  |  |  |  |
| Lives with both parents | 119063 (82.8) | 297 (91.7) | 261 (78.4) | 269 (76.6) | 80 (54.1) | 89 (64.0) | <.0001 |
| Lives with only mother | 14064 (9.8) | 20 (6.2) | 41 (12.3) | 43 (12.3) | 33 (22.3) | 23 (16.5) |  |
| Lives with only father | 6338 (4.4) | 4 (1.2) | 20 (6.0) | 23 (6.6) | 17 (11.5) | 4 (2.9) |  |
| Lives with no parents | 4344 (3.0) | 3 (0.9) | 11 (3.3) | 16 (4.6) | 18 (12.2) | 23 (16.5) |  |

Table S4. Age-adjusted prevalence (95% confidence intervals) of sadness/despair and suicidal ideation among boys and girls, according to socioeconomic position indicators.

|  | **Depressive mood** | | Suicidal ideation | |
| --- | --- | --- | --- | --- |
|  | Boys | Girls | Boys | Girls |
| **Urbanity** |  |  |  |  |
| Metropolitan cities | 25.6 (25.1–26.0) | 35.9 (35.4–36.5) | 13.6 (13.2–13.9) | 20.8 (20.4–21.3) |
| Small and medium cities | 24.8 (24.4–25.2) | 35.3 (34.8–35.8) | 13.0 (12.6–13.4) | 20.3 (19.8–20.8) |
| Rural areas | 25.9 (25.0–26.8) | 38.3 (37.1–39.6) | 13.3 (12.6–14.0) | 20.7 (19.8–21.7) |
| P-value | 0.0215 | <.0001 | 0.0777 | 0.2974 |
| **Father’s education** |  |  |  |  |
| College | 28.3 (27.0–29.7) | 40.1 (38.7–41.6) | 15.5 (14.4–16.6) | 26.2 (24.9–27.4) |
| High school | 25.0 (24.5–25.5) | 36.2 (35.7–36.7) | 12.6 (12.2–13.0) | 20.9 (20.4–21.4) |
| Middle school | 25.5 (25.1–26.0) | 34.8 (34.4–35.3) | 13.6 (13.3–13.9) | 19.7 (19.3–20.2) |
| Do not know | 22.7 (22.1–23.4) | 34.6 (33.8–35.4) | 12.1 (11.6–12.6) | 19.2 (18.6–19.9) |
| No father | 29.2 (27.9–30.5) | 43.4 (41.9–44.9) | 17.0 (15.9–18.1) | 27.2 (25.9–28.5) |
| P-value | <.0001 | <.0001 | <.0001 | <.0001 |
| P for trend* | <.0001 | <.0001 | <.0001 | <.0001 |
| **Mother’s education** |  |  |  |  |
| College | 27.9 (26.5–29.3) | 40.2 (38.7–41.7) | 16.0 (14.9–17.2) | 25.0 (23.7–26.3) |
| High school | 25.0 (24.5–25.4) | 35.6 (35.1–36.1) | 12.6 (12.3–12.9) | 20.5 (20.1–20.9) |
| Middle school | 25.6 (25.2–26.1) | 35.1 (34.6–35.6) | 13.6 (13.3–14.0) | 19.9 (19.4–20.3) |
| Do not know | 22.6 (22.0–23.3) | 34.4 (33.6–35.3) | 12.0 (11.5–12.5) | 19.4 (18.7–20.1) |
| No mother | 31.4 (30.0–32.8) | 46.3 (44.7–47.9) | 19.1 (18.0–20.3) | 29.2 (27.7–30.7) |
| P-value | <.0001 | <.0001 | <.0001 | <.0001 |
| P for trend* | 0.0006 | <.0001 | <.0001 | <.0001 |
| **Perceived household economic status** |  |  |  |  |
| High | 25.5 (24.7–26.3) | 33.0 (31.8–34.2) | 14.2 (13.5–14.9) | 18.3 (17.2–19.4) |
| Mid-high | 23.6 (23.1–24.2) | 33.1 (32.5–33.7) | 12.1 (11.7–12.5) | 17.8 (17.3–18.3) |
| Middle | 23.1 (22.7–23.5) | 33.4 (33.0–33.9) | 11.4 (11.1–11.7) | 18.3 (18.0–18.7) |
| Mid-low | 29.5 (28.8–30.2) | 42.6 (41.9–43.3) | 16.4 (15.8–16.9) | 27.1 (26.4–27.7) |
| Low | 38.6 (37.3–39.9) | 54.1 (52.7–55.6) | 25.4 (24.3–26.5) | 38.9 (37.5–40.3) |
| P-value | <.0001 | <.0001 | <.0001 | <.0001 |
| P for trend | <.0001 | <.0001 | <.0001 | <.0001 |
| **Self-reported educational performance** |  |  |  |  |
| High | 21.3 (20.6–22.0) | 28.0 (27.1–28.8) | 12.4 (11.8–13.0) | 17.0 (16.3–17.7) |
| Mid-high | 22.8 (22.2–23.3) | 30.8 (30.2–31.3) | 11.8 (11.4–12.2) | 17.6 (17.1–18.1) |
| Middle | 23.4 (22.9–23.9) | 33.2 (32.7–33.8) | 11.9 (11.5–12.3) | 17.9 (17.5–18.4) |
| Mid-low | 27.6 (27.1–28.2) | 40.5 (39.9–41.1) | 14.2 (13.8–14.6) | 23.3 (22.8–23.8) |
| Low | 32.7 (31.9–33.5) | 49.2 (48.4–50.1) | 18.0 (17.4–18.7) | 30.7 (29.8–31.5) |
| P-value | <.0001 | <.0001 | <.0001 | <.0001 |
| P for trend | <.0001 | <.0001 | <.0001 | <.0001 |
| **Cohabitation with parents** |  |  |  |  |
| Lives with both parents | 24.5 (24.2–24.8) | 34.4 (34.1–34.8) | 12.8 (12.5–13.0) | 19.4 (19.0–19.7) |
| Lives with only mother | 27.8 (26.9–28.7) | 40.7 (39.8–41.7) | 14.3 (13.6–15.0) | 25.1 (24.2–25.9) |
| Lives with only father | 29.3 (28.1–30.5) | 44.5 (43.0–45.9) | 16.1 (15.2–17.1) | 28.6 (27.3–29.9) |
| Lives with no parents | 31.3 (29.7–32.9) | 47.4 (45.6–49.3) | 19.6 (18.3–21.0) | 30.6 (29.0–32.3) |
| P-value | <.0001 | <.0001 | <.0001 | <.0001 |

*Excluding responses of do not know, no father, or no mother.

Table S5. Age-adjusted prevalence (95% confidence intervals) of suicidal plans and suicide attempts among boys and girls, according to socioeconomic position indicators.

|  | Suicidal plans | | Suicide attempts | |
| --- | --- | --- | --- | --- |
|  | Boys | Girls | Boys | Girls |
| **Urbanity** |  |  |  |  |
| Metropolitan cities | 4.9 (4.7–5.1) | 6.7 (6.5–7.0) | 2.6 (2.5–2.8) | 4.8 (4.6–5.0) |
| Small and medium cities | 4.6 (4.4–4.8) | 6.6 (6.3–6.8) | 2.5 (2.4–2.7) | 5.0 (4.7–5.2) |
| Rural areas | 5.2 (4.7–5.7) | 6.8 (6.2–7.4) | 3.1 (2.7–3.4) | 5.0 (4.5–5.5) |
| P-value | 0.0198 | 0.5577 | 0.0105 | 0.452 |
| **Father’s education** |  |  |  |  |
| College | 6.0 (5.3–6.8) | 8.5 (7.7–9.3) | 3.8 (3.2–4.3) | 6.8 (6.1–7.6) |
| High school | 4.3 (4.1–4.5) | 6.6 (6.3–6.8) | 2.4 (2.2–2.5) | 4.9 (4.7–5.2) |
| Middle school | 4.8 (4.6–5.0) | 6.3 (6.0–6.5) | 2.5 (2.4–2.7) | 4.4 (4.2–4.6) |
| Do not know | 4.4 (4.1–4.7) | 6.3 (5.9–6.7) | 2.5 (2.2–2.7) | 4.7 (4.3–5.0) |
| No father | 7.7 (7.0–8.5) | 11.1 (10.1–12.1) | 5.3 (4.6–5.9) | 8.7 (7.9–9.5) |
| P-value | <.0001 | <.0001 | <.0001 | <.0001 |
| P for trend* | <.0001 | <.0001 | <.0001 | <.0001 |
| **Mother’s education** |  |  |  |  |
| College | 5.8 (5.0–6.5) | 8.4 (7.6–9.2) | 2.8 (2.3–3.3) | 6.2 (5.5–6.9) |
| High school | 4.2 (4.0–4.4) | 6.4 (6.1–6.6) | 2.3 (2.2–2.5) | 4.7 (4.5–4.9) |
| Middle school | 4.9 (4.7–5.2) | 6.4 (6.2–6.7) | 2.6 (2.5–2.8) | 4.5 (4.3–4.7) |
| Do not know | 4.3 (4.0–4.6) | 6.4 (6.0–6.8) | 2.6 (2.3–2.8) | 4.9 (4.5–5.3) |
| No mother | 9.5 (8.6–10.4) | 12.3 (11.2–13.3) | 5.9 (5.2–6.6) | 10.5 (9.5–11.5) |
| P-value | <.0001 | <.0001 | <.0001 | <.0001 |
| P for trend* | 0.0061 | <.0001 | 0.2926 | <.0001 |
| **Perceived household economic status** |  |  |  |  |
| High | 7.0 (6.5–7.4) | 7.1 (6.4–7.8) | 4.2 (3.9–4.6) | 4.9 (4.3–5.6) |
| Mid-high | 4.1 (3.9–4.4) | 5.6 (5.3–5.9) | 2.1 (1.9–2.3) | 3.9 (3.6–4.1) |
| Middle | 3.8 (3.6–4.0) | 5.6 (5.4–5.8) | 2.0 (1.9–2.2) | 4.1 (3.9–4.3) |
| Mid-low | 5.2 (4.9–5.5) | 8.7 (8.3–9.1) | 2.8 (2.5–3.0) | 6.4 (6.1–6.8) |
| Low | 11.4 (10.6–12.2) | 16.2 (15.1–17.3) | 7.5 (6.8–8.2) | 12.8 (11.9–13.8) |
| P-value | <.0001 | <.0001 | <.0001 | <.0001 |
| P for trend | <.0001 | <.0001 | <.0001 | <.0001 |
| **Self-reported educational performance** |  |  |  |  |
| High | 4.9 (4.5–5.3) | 5.1 (4.7–5.5) | 2.8 (2.5–3.0) | 3.1 (2.7–3.4) |
| Mid-high | 4.0 (3.7–4.2) | 5.5 (5.2–5.8) | 2.0 (1.9–2.2) | 3.6 (3.3–3.8) |
| Middle | 4.1 (3.8–4.3) | 5.3 (5.1–5.6) | 2.1 (1.9–2.2) | 3.8 (3.6–4.0) |
| Mid-low | 4.9 (4.6–5.1) | 7.6 (7.3–7.9) | 2.6 (2.4–2.8) | 6.0 (5.7–6.3) |
| Low | 7.3 (6.9–7.7) | 11.6 (11.0–12.2) | 4.8 (4.5–5.2) | 9.5 (9.0–10.1) |
| P-value | <.0001 | <.0001 | <.0001 | <.0001 |
| P for trend | <.0001 | <.0001 | <.0001 | <.0001 |
| **Cohabitation with parents** |  |  |  |  |
| Lives with both parents | 4.5 (4.3–4.6) | 6.0 (5.8–6.2) | 2.4 (2.3–2.5) | 4.3 (4.2–4.5) |
| Lives with only mother | 5.1 (4.7–5.5) | 8.9 (8.4–9.5) | 2.9 (2.5–3.2) | 6.7 (6.2–7.1) |
| Lives with only father | 6.2 (5.5–6.8) | 10.7 (9.8–11.6) | 3.7 (3.2–4.2) | 8.8 (8.0–9.5) |
| Lives with no parents | 9.3 (8.2–10.3) | 13.0 (11.8–14.2) | 6.3 (5.5–7.2) | 10.4 (9.3–11.5) |
| P-value | <.0001 | <.0001 | <.0001 | <.0001 |

*Excludes responses of do not know, no father, or no mother.

Table S6. PRs and percentage reduction in PRs of depressive mood and suicidality among Korean boys and girls by multicultural status.

|  | Boys |  | Girls |  |
| --- | --- | --- | --- | --- |
|  | PR | % reduction in PRs (%) | PR | % reduction in PRs (%) |
| **Depressive mood** |  |  |  |  |
| Baseline model (adjusted for age) | 1.12 |  | 1.05 |  |
| + Urbanity | 1.12 | 0.0 | 1.04 | 20.0 |
| + Father’s education | 1.12 | 0.0 | 1.02 | 60.0 |
| + Mother’s education | 1.13 | -8.3 | 1.05 | 0.0 |
| + Perceived household economic status | 1.07 | 41.7 | 0.99 | 120.0 |
| + Self-reported educational performance | 1.1 | 16.7 | 1 | 100.0 |
| + Cohabitation with parents | 1.1 | 16.7 | 1.03 | 40.0 |
| + All socioeconomic position indicators | 1.07 | 41.7 | 0.98 | 140.0 |
| **Suicidal ideation** |  |  |  |  |
| Baseline model (adjusted for age) | 1.33 |  | 0.98 |  |
| + Urbanity | 1.33 | 0.0 | 0.98 | 0.0 |
| + Father’s education | 1.31 | 6.1 | 0.94 | -200.0 |
| + Mother’s education | 1.33 | 0.0 | 0.98 | 0.0 |
| + Perceived household economic status | 1.21 | 36.4 | 0.91 | -350.0 |
| + Self-reported educational performance | 1.28 | 15.2 | 0.94 | -200.0 |
| + Cohabitation with parents | 1.29 | 12.1 | 0.96 | -100.0 |
| + All socioeconomic position indicators | 1.21 | 36.4 | 0.9 | -400.0 |
| **Suicidal plans** |  |  |  |  |
| Baseline model (adjusted for age) | 1.74 |  | 1.16 |  |
| + Urbanity | 1.73 | 1.4 | 1.16 | 0.0 |
| + Father’s education | 1.69 | 6.8 | 1.08 | 50.0 |
| + Mother’s education | 1.75 | -1.4 | 1.16 | 0.0 |
| + Perceived household economic status | 1.57 | 23.0 | 1.05 | 68.8 |
| + Self-reported educational performance | 1.67 | 9.5 | 1.08 | 50.0 |
| + Cohabitation with parents | 1.65 | 12.2 | 1.1 | 37.5 |
| + All socioeconomic position indicators | 1.52 | 29.7 | 1.03 | 81.3 |
| **Suicide attempts** |  |  |  |  |
| Baseline model (adjusted for age) | 2.6 |  | 1.28 |  |
| + Urbanity | 2.56 | 2.5 | 1.28 | 0.0 |
| + Father’s education | 2.45 | 9.4 | 1.17 | 39.3 |
| + Mother’s education | 2.6 | 0.0 | 1.28 | 0.0 |
| + Perceived household economic status | 2.26 | 21.3 | 1.15 | 46.4 |
| + Self-reported educational performance | 2.43 | 10.6 | 1.17 | 39.3 |
| + Cohabitation with parents | 2.41 | 11.9 | 1.21 | 25.0 |
| + All socioeconomic position indicators | 2.11 | 30.6 | 1.11 | 60.7 |

Table S7. PRs and percentage reduction in PRs of depressive mood and suicidality among Korean boys by parental country of birth.

|  | Japan-born mother | | Korean-Chinese mother | | Other foreign-born mother | | Foreign-born father | | Both parents foreign-born | |
| --- | --- | --- | --- | --- | --- | --- | --- | --- | --- | --- |
|  | PR | % reduction in PRs | PR | % reduction in PRs | PR | % reduction in PRs | PR | % reduction in PRs | PR | % reduction in PRs |
| **Depressive mood** |  |  |  |  |  |  |  |  |  |  |
| Baseline model (adjusted for age) | 0.84 |  | 1.00 |  | 1.09 |  | 1.62 |  | 1.32 |  |
| + Urbanity | 0.84 | 0.0 | 1.00 | NA | 1.09 | 0.0 | 1.62 | 0.0 | 1.32 | 0.0 |
| + Father’s education | 0.83 | -6.3 | 0.97 | NA | 1.09 | 0.0 | 1.65 | -4.8 | 1.32 | 0.0 |
| + Mother’s education | 0.85 | 6.3 | 1.02 | NA | 1.12 | -33.3 | 1.52 | 16.1 | 1.33 | -3.1 |
| + Perceived household economic status | 0.81 | -18.7 | 0.94 | NA | 1.04 | 55.6 | 1.49 | 21.0 | 1.22 | 31.3 |
| + Self-reported educational performance | 0.83 | -6.3 | 0.96 | NA | 1.04 | 55.6 | 1.57 | 8.1 | 1.27 | 15.6 |
| + Cohabitation with parents | 0.85 | 6.3 | 0.98 | NA | 1.07 | 22.2 | 1.51 | 17.7 | 1.27 | 15.6 |
| + All socioeconomic position indicators | 0.82 | -12.5 | 0.95 | NA | 1.05 | 44.4 | 1.41 | 33.9 | 1.19 | 40.6 |
| **Suicidal ideation** |  |  |  |  |  |  |  |  |  |  |
| Baseline model (adjusted for age) | 0.74 |  | 0.93 |  | 1.33 |  | 2.01 |  | 2.22 |  |
| + Urbanity | 0.74 | 0.0 | 0.93 | 0.0 | 1.33 | 0.0 | 2.01 | 0.0 | 2.22 | 0.0 |
| + Father’s education | 0.74 | 0.0 | 0.90 | -42.9 | 1.32 | 3.0 | 2.03 | -2.0 | 2.21 | 0.8 |
| + Mother’s education | 0.75 | 3.8 | 0.95 | 28.6 | 1.37 | -12.1 | 1.79 | 21.8 | 2.21 | 0.8 |
| + Perceived household economic status | 0.71 | -11.5 | 0.85 | -114.3 | 1.25 | 24.2 | 1.73 | 27.7 | 1.93 | 23.8 |
| + Self-reported educational performance | 0.74 | 0.0 | 0.90 | -42.9 | 1.27 | 18.2 | 1.91 | 9.9 | 2.08 | 11.5 |
| + Cohabitation with parents | 0.75 | 3.8 | 0.91 | -28.6 | 1.30 | 9.1 | 1.80 | 20.8 | 2.07 | 12.3 |
| + All socioeconomic position indicators | 0.73 | -3.8 | 0.88 | -71.4 | 1.27 | 18.2 | 1.57 | 43.6 | 1.82 | 32.8 |
| **Suicidal plans** |  |  |  |  |  |  |  |  |  |  |
| Baseline model (adjusted for age) | 0.34 |  | 0.66 |  | 1.95 |  | 3.47 |  | 3.86 |  |
| + Urbanity | 0.33 | -1.5 | 0.66 | 0.0 | 1.94 | 1.1 | 3.47 | 0.0 | 3.85 | 0.3 |
| + Father’s education | 0.33 | -1.5 | 0.62 | -11.8 | 1.89 | 6.3 | 3.51 | -1.6 | 3.78 | 2.8 |
| + Mother’s education | 0.35 | 1.5 | 0.70 | 11.8 | 2.05 | -10.5 | 2.74 | 29.6 | 3.88 | -0.7 |
| + Perceived household economic status | 0.33 | -1.5 | 0.61 | -14.7 | 1.82 | 13.7 | 2.69 | 31.6 | 2.96 | 31.5 |
| + Self-reported educational performance | 0.34 | 0.0 | 0.64 | -5.9 | 1.84 | 11.6 | 3.18 | 11.7 | 3.44 | 14.7 |
| + Cohabitation with parents | 0.35 | 1.5 | 0.64 | -5.9 | 1.86 | 9.5 | 2.91 | 22.7 | 3.37 | 17.1 |
| + All socioeconomic position indicators | 0.34 | 0.0 | 0.64 | -5.9 | 1.86 | 9.5 | 2.27 | 48.6 | 2.78 | 37.8 |
| **Suicide attempts** |  |  |  |  |  |  |  |  |  |  |
| Baseline model (adjusted for age) | 0.84 |  | 0.65 |  | 2.97 |  | 5.16 |  | 5.45 |  |
| + Urbanity | 0.82 | -12.5 | 0.64 | -2.9 | 2.92 | 2.5 | 5.16 | 0.0 | 5.45 | 0.0 |
| + Father’s education | 0.81 | -18.7 | 0.57 | -22.9 | 2.75 | 11.2 | 5.26 | -2.4 | 5.26 | 4.3 |
| + Mother’s education | 0.87 | 18.8 | 0.68 | 8.6 | 3.10 | -6.6 | 3.90 | 30.3 | 5.60 | -3.4 |
| + Perceived household economic status | 0.82 | -12.5 | 0.59 | -17.1 | 2.73 | 12.2 | 3.73 | 34.4 | 3.87 | 35.5 |
| + Self-reported educational performance | 0.85 | 6.3 | 0.63 | -5.7 | 2.73 | 12.2 | 4.52 | 15.4 | 4.58 | 19.6 |
| + Cohabitation with parents | 0.88 | 25.0 | 0.62 | -8.6 | 2.78 | 9.6 | 4.02 | 27.4 | 4.49 | 21.6 |
| + All socioeconomic position indicators | 0.83 | -6.3 | 0.58 | -20.0 | 2.64 | 16.8 | 3.15 | 48.3 | 3.47 | 44.5 |

Table S8. PRs and percentage reduction in PRs of depressive mood and suicidality among Korean girls by parental country of birth.

|  | Japan-born mother | | Korean-Chinese mother | | Other foreign-born mother | | Foreign-born father | | Both parents foreign-born | |
| --- | --- | --- | --- | --- | --- | --- | --- | --- | --- | --- |
|  | PR | % reduction in PRs | PR | % reduction in PRs | PR | % reduction in PRs | PR | % reduction in PRs | PR | % reduction in PRs |
| **Depressive mood** |  |  |  |  |  |  |  |  |  |  |
| Baseline model (adjusted for age) | 0.77 |  | 1.01 |  | 1.12 |  | 1.29 |  | 1.35 |  |
| + Urbanity | 0.77 | 0.0 | 1.00 | 100.0 | 1.11 | 8.3 | 1.29 | 0.0 | 1.35 | 0.0 |
| + Father’s education | 0.77 | 0.0 | 0.97 | 400.0 | 1.08 | 33.3 | 1.30 | -3.4 | 1.34 | 2.9 |
| + Mother’s education | 0.79 | 8.7 | 1.01 | 0.0 | 1.14 | -16.7 | 1.25 | 13.8 | 1.34 | 2.9 |
| + Perceived household economic status | 0.74 | -13.0 | 0.97 | 400.0 | 1.08 | 33.3 | 1.22 | 24.1 | 1.29 | 17.1 |
| + Self-reported educational performance | 0.77 | 0.0 | 0.96 | 500.0 | 1.06 | 50.0 | 1.22 | 24.1 | 1.33 | 5.7 |
| + Cohabitation with parents | 0.79 | 8.7 | 0.99 | 200.0 | 1.10 | 16.7 | 1.22 | 24.1 | 1.26 | 25.7 |
| + All socioeconomic position indicators | 0.74 | -13.0 | 0.96 | 500.0 | 1.03 | 75.0 | 1.14 | 51.7 | 1.27 | 22.9 |
| **Suicidal ideation** |  |  |  |  |  |  |  |  |  |  |
| Baseline model (adjusted for age) | 0.59 |  | 1.05 |  | 1.11 |  | 1.06 |  | 1.41 |  |
| + Urbanity | 0.59 | 0.0 | 1.05 | 0.0 | 1.11 | 0.0 | 1.06 | 0.0 | 1.41 | 0.0 |
| + Father’s education | 0.57 | -4.9 | 0.98 | 140.0 | 1.03 | 72.7 | 1.08 | -33.3 | 1.38 | 7.3 |
| + Mother’s education | 0.60 | 2.4 | 1.04 | 20.0 | 1.12 | -9.1 | 1.03 | 50.0 | 1.38 | 7.3 |
| + Perceived household economic status | 0.54 | -12.2 | 0.98 | 140.0 | 1.03 | 72.7 | 0.97 | 150.0 | 1.30 | 26.8 |
| + Self-reported educational performance | 0.58 | -2.4 | 0.99 | 120.0 | 1.04 | 63.6 | 1.00 | 100.0 | 1.37 | 9.8 |
| + Cohabitation with parents | 0.60 | 2.4 | 1.02 | 60.0 | 1.07 | 36.4 | 0.98 | 133.3 | 1.27 | 34.1 |
| + All socioeconomic position indicators | 0.56 | -7.3 | 0.97 | 160.0 | 0.99 | 109.1 | 0.90 | 266.7 | 1.23 | 43.9 |
| **Suicidal plans** |  |  |  |  |  |  |  |  |  |  |
| Baseline model (adjusted for age) | 0.48 |  | 1.12 |  | 1.03 |  | 1.57 |  | 3.20 |  |
| + Urbanity | 0.48 | 0.0 | 1.12 | 0.0 | 1.03 | 0.0 | 1.57 | 0.0 | 3.20 | 0.0 |
| + Father’s education | 0.47 | -1.9 | 1.01 | 91.7 | 0.94 | 300.0 | 1.61 | -7.0 | 3.11 | 4.1 |
| + Mother’s education | 0.49 | 1.9 | 1.12 | 0.0 | 1.05 | -66.7 | 1.47 | 17.5 | 3.09 | 5.0 |
| + Perceived household economic status | 0.44 | -7.7 | 1.04 | 66.7 | 0.95 | 266.7 | 1.38 | 33.3 | 2.70 | 22.7 |
| + Self-reported educational performance | 0.48 | 0.0 | 1.04 | 66.7 | 0.95 | 266.7 | 1.45 | 21.1 | 3.05 | 6.8 |
| + Cohabitation with parents | 0.50 | 3.8 | 1.07 | 41.7 | 0.97 | 200.0 | 1.38 | 33.3 | 2.64 | 25.5 |
| + All socioeconomic position indicators | 0.47 | -1.9 | 1.03 | 75.0 | 0.90 | 433.3 | 1.24 | 57.9 | 2.48 | 32.7 |
| **Suicide attempt** |  |  |  |  |  |  |  |  |  |  |
| Baseline model (adjusted for age) | 0.60 |  | 1.40 |  | 1.05 |  | 1.88 |  | 3.01 |  |
| + Urbanity | 0.60 | 0.0 | 1.39 | 2.5 | 1.04 | 20.0 | 1.89 | -1.1 | 3.01 | 0.0 |
| + Father’s education | 0.57 | -7.5 | 1.22 | 45.0 | 0.92 | 260.0 | 1.95 | -8.0 | 2.85 | 8.0 |
| + Mother’s education | 0.63 | 7.5 | 1.39 | 2.5 | 1.07 | -40.0 | 1.73 | 17.0 | 2.90 | 5.5 |
| + Perceived household economic status | 0.54 | -15.0 | 1.28 | 30.0 | 0.96 | 180.0 | 1.64 | 27.3 | 2.51 | 24.9 |
| + Self-reported educational performance | 0.59 | -2.5 | 1.26 | 35.0 | 0.94 | 220.0 | 1.68 | 22.7 | 2.84 | 8.5 |
| + Cohabitation with parents | 0.63 | 7.5 | 1.32 | 20.0 | 0.98 | 140.0 | 1.63 | 28.4 | 2.42 | 29.4 |
| + All socioeconomic position indicators | 0.58 | -5.0 | 1.25 | 37.5 | 0.87 | 360.0 | 1.44 | 50.0 | 2.31 | 34.8 |
